# Aberrant Learned Irrelevance in Patients with First-Episode Schizophrenia-Spectrum Disorder

**DOI:** 10.3390/brainsci11111370

**Published:** 2021-10-20

**Authors:** Ryan Sai-Ting Chu, Chung-Mun Ng, Kwun-Nam Chan, Kit-Wa Chan, Ho-Ming Lee, Lai-Ming Hui, Eric Chen, Wing-Chung Chang

**Affiliations:** 1 Department of Psychiatry, Queen Mary Hospital, The University of Hong Kong, Hong Kong; ryancst@hotmail.com (R.S.-T.C.); maryncm@hku.hk (C.-M.N.); chankwunnam@gmail.com (K.-N.C.); kwsherry@hku.hk (K.-W.C.); edwinlhm@hku.hk (H.-M.L.); christyh@hku.hk (L.-M.H.); eyhchen@hku.hk (E.C.); 2 State Key Laboratory of Brain and Cognitive Sciences, The University of Hong Kong, Hong Kong

**Keywords:** learned irrelevance, selective attention, positive symptoms, first-episode psychosis, schizophrenia

## Abstract

Emerging evidence has indicated disrupted learned irrelevance (LIrr), a form of selective attention deficit that may contribute to psychotic symptom formation, in schizophrenia. However, previous research mostly focused on chronic patients. There is a paucity of studies on LIrr in first-episode schizophrenia-spectrum disorder (i.e., schizophrenia and schizophreniform disorder; FES), which were limited by small sample size and have produced mixed results. The current study examined a LIrr effect and its relationship with positive symptom severity in 40 briefly-medicated FES patients and 42 demographically-matched healthy controls using a well-validated computerized LIrr paradigm which has been applied in chronic schizophrenia sample. Positive symptoms were assessed by Positive and Negative Syndrome Scale (PANSS) and Psychotic Symptom Rating Scales (PSYRATS). Our results showed that controls demonstrated intact LIrr, with significantly faster learning about previously predictive (relevant) than previously non-predictive (irrelevant) cues. Lack of such normal attention bias towards predictive over non-predictive cues was observed in FES patients, indicating their failure to distinguish between relevant and irrelevant stimuli. Nonetheless, we failed to reveal any significant correlations between learning scores, in particular learning scores for non-predictive cues, and positive symptom measures in FES patients. Learning scores were also not associated with other symptom dimensions, cognitive functions and antipsychotic dose. In conclusion, our findings indicate aberrant LIrr with impaired allocation of attention to relevant versus irrelevant stimuli in briefly-medicated FES patients. Further prospective research is warranted to clarify the longitudinal trajectory of such selective attention deficit and its association with positive symptoms and treatment response in the early course of illness.

## 1. Introduction

Substantial evidence indicates that schizophrenia is associated with deficient selective attention [1,2], which refers to an inability to adequately allocate attention to relevant stimuli while ignoring irrelevant stimuli in the environment. Previous research has often adopted a latent inhibition (LI) paradigm to examine selective attention deficits in schizophrenia [3,4]. LI denotes retardation in learning of stimulus–outcome association following pre-exposure to that stimulus without consequence [5]. Some, though not all, of these prior studies have reported attenuated LI in schizophrenia, consistent with a deficit in the ability to learn to ignore inconsequential stimuli [3,4,6].

Recently, learned irrelevance (LIrr), a pre-exposure effect closely related to LI, has been put forward as a more sensitive and less ambiguous measure of the influence of selective attention deficits on learning in schizophrenia [7,8]. In an LIrr procedure, participants undergo initial training on a task in which certain stimuli are experienced as irrelevant with regard to outcomes—these stimuli do not predict which outcome will occur—whereas other stimuli are experienced as predictive of which outcome will occur. Healthy participants are slower to learn novel associations regarding cues previously established as non-predictive than those established as predictive (the LIrr effect). By contrast, accumulating data have shown that schizophrenia patients exhibited disrupted LIrr [9,10,11], suggesting their impaired ability to reduce attention to irrelevant stimuli compared with healthy controls. Importantly, such dysfunction aligns with the aberrant salience model of psychosis [12,13], which postulates that striatal dopamine dysregulation causes misattribution of salience to irrelevant stimuli, resulting in formation of psychotic symptoms [14,15]. In fact, there is evidence suggesting that positive psychotic symptoms are correlated with altered attention to irrelevant stimuli in schizophrenia patients [16]. Alternatively, some prior studies have also explored the relationship between abnormal LIrr and other symptom dimensions but yielded mixed results. For instance, one recent study suggested that high level of negative symptom dimension of schizotypy might be associated with reduced normal attentional bias towards relevant stimuli [17], while another report revealed significant correlations between depressive symptoms and LIrr task performance [11]. Whether LIrr process might be adversely affected by impairment in general cognitive functions remains unknown.

Of note, prior studies on LIrr in schizophrenia mainly focused on patients with chronic illness. Very few studies have been conducted to examine first-episode samples in this respect and were limited by small sample size (*n* < 15) [9,11]. It is also suggested that some earlier experimental paradigms may not clearly disentangle the LIrr effect from other alternative, non-attentional explanations, such as learning impairment, contributing to abnormal task performance [8]. In the current study, we sought to investigate LIrr in a cohort of first-episode schizophrenia-spectrum disorder (including schizophrenia and schizophreniform disorder; FES) patients using a modified version of Le Pelley and McLaren’s LIrr paradigm [18], which has been studied in chronic schizophrenia [16] and was designed to reliably detect and quantify disrupted selective attention. Specifically, we aimed to assess whether FES patients would fail to discriminate between predictive (relevant) and non-predictive (irrelevant) cues (i.e., absence of normal retarded LIrr effect) relative to healthy controls, and to examine whether new learning about nonpredictive cues (an index of attention to irrelevant stimuli) would be associated with positive psychotic symptoms. We also explored the associations of LIrr task performance with other symptom dimensions and cognitive functions.

## 2. Materials and Methods

### 2.1. Participants

Forty patients in their first psychotic episode, aged 15–40 years, were recruited from the outpatient unit of a specialized early intervention service for first-episode psychosis [19] in Hong Kong (HK). Diagnosis of schizophrenia or schizophreniform disorder was ascertained at intake using the Chinese-bilingual Structured Clinical Interview for DSM-IV (CB-SCID-I/P) [20,21] and medical record review. We combined schizophrenia and schizophreniform disorder into a single diagnostic category of schizophrenia-spectrum disorder for analysis because substantial evidence has indicated that schizophreniform disorder is diagnostically unstable and the vast majority of patients with this initial diagnosis switch to schizophrenia diagnosis at follow-up [22,23]. Interview for the Retrospective Assessment of the Onset of Schizophrenia (IRAOS) [24] was used to verify first-episode status and assess duration of untreated psychosis. Study assessments were administered to patients within three months following antipsychotic initiation (median: 22 days). Forty-two demographically-matched healthy controls were recruited from the community via advertisements for comparison. Controls were screened to confirm that they had no psychiatric diagnosis (by CB-SCID-I/P), family history of psychotic disorder, and were not taking any psychotropic medications. Any individual with history of alcohol or substance abuse (according to the Alcohol Use Scale and the Drug Use Scale) [25], intellectual disability or neurological diseases was excluded from participation. The study was approved by the local institutional review boards, and all participants provided written informed consent. For those aged under 18 years, parental consent was also obtained.

### 2.2. Clinical and Cognitive Assessments

Patients’ positive psychotic symptoms were assessed using the Positive and Negative Syndrome Scale (PANSS) [26] and the Psychotic Symptom Rating Scales (PSYRATS) [27]. Specifically, PANSS positive symptom dimension score derived on the basis of a previous factor-analytic study in early psychosis sample [28] was adopted to measure positive symptom severity. PSYRATS total score as well as delusion and hallucination subscale scores were also used to assess positive symptom levels. The Brief Negative Symptom Scale (BNSS) [29] and the Calgary Depression Scale for Schizophrenia (CDSS) [30] were applied to assess negative and depressive symptoms, respectively. A brief standardized cognitive battery was administered to both patients and controls, including letter-number-span [31] for working memory, digit symbol subtest from the Wechsler Adult Intelligence Scale-Revised (WAIS-R) [32] for processing speed, logical memory subtest from the Wechsler Adult Memory Scale-Revised (WMS-R) [33] for episodic memory, letter-cancellation test [34] for sustained attention, and trail-making test [35] for attention and set-shifting executive functions. 

### 2.3. Learned Irrelevance Task

**Figure 1:**
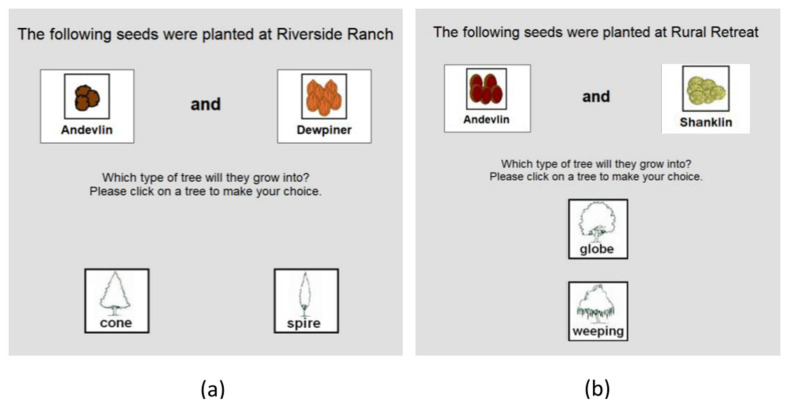
Schematic illustration with screenshots of the learned irrelevance (LIrr) task (adapted from Morris et al., 2013 [16]). The instruction is shown in traditional Chinese. (**a**) Sample screen capture for Stage 1. Participants were instructed to make predictions about which combination of seed varieties (Cues A or B paired with V or W) would produce which type of tree (Outcomes o1 or o2) on a farm called Riverside Ranch, resulting in four cue-outcome combinations (AV–o1, AW–o1, BV–o2 and BW–o2). Feedback of “correct” or “incorrect” was provided after each prediction. (**b**) Sample screen capture for Stage 2. Participants were instructed to work on a new farm, Rural Retreat, and to learn to predict 2 new types of tree (Outcomes o3 or o4) using the same seed varieties as in Stage 1 but with two cue-outcome combinations (AV-o3, BW-o4). No feedback was provided. Cues A and B represented previously predictive cues, while Cues V and W denoted previously non-predictive cues. A LIrr effect was demonstrated if a participant learned more about predictive over non-predictive cues during Stage 2, indicating normal attention bias towards previously predictive over non-predictive cues (i.e., retarded learning about previously non-predictive cues).

A computerized learned irrelevance (LIrr) task was adapted from the experimental paradigm used in used in Experiment 2 of Morris et al. [16] for studying patients with chronic schizophrenia. Schematic illustration of the LIrr task is shown in Figure 1. In brief, all participants were instructed to act as a horticulturalist to develop new plant species in different virtual farms. In Stage 1, participants were asked to make predictions about which combination of seed varieties (Cues A or B paired with V or W) (i.e., Dewpiner or Andevlin with Millerbob or Shanklin as displayed in the screen) would produce which type of tree (Outcomes o1 or o2) (i.e., cone or spire) on a farm (Riverside Ranch). Participants would receive feedback after each prediction; The word “correct” would appear on a computer screen when the right tree was selected and the word “incorrect” for selecting the wrong tree. There were 20 blocks in total, with 4 cue-outcome combination trials (AV–o1, AW–o1, BV–o2 and BW–o2) shown in random order in each block. To ensure participants learned the relevant cue-outcome relationship in Stage 1 and that the reduced LIrr performance with absence of bias towards previously predictive cues (learned in Stage 1) in Stage 2 could not be explained by deficit in learning, 6 consecutive correct trials had to be achieved before participants could progress to Stage 2. In Stage 2, participants were instructed to work in a new farm (rural retreat) and to learn to predict two new types of tree (Outcomes o3 or o4) (i.e., globe or weeping) using the same seed varieties. There were 8 blocks in total, with 2 cue-outcome combination trials (AV–o3 and BW–o4) displayed in random order in each block. No feedback was provided in stage 2. Participants were then asked to take 2 tests to illustrate what they had learned in stage 2. In each test, a combination of 8 trials was conducted for each outcome (o3 and o4) per individual cue (A, B, V, and W) in random order. For each trial, participants were asked to rate the level of confidence of the selected cue-outcome combination on a scale from 0 (very unlikely) to 10 (very likely). The learning score per cue of each participant was generated using a confidence rating based on the cue-outcome combination. If the corresponding outcome was correct, the confidence rating would be multiplied by 1 whereas if the corresponding outcome was incorrect, the confidence rating would be multiplied by −1. Thus, the learning score ranged from −10 to 10. Lower scores suggested participants having higher level of confidence in wrong cue-outcome combination while higher scores indicated participants having higher level of confidence in correct cue-outcome combination. The mean of learning scores for Cues A and B was utilized as the score for the *previously predictive cues*, whereas that for Cues V and W was utilized as the score for the *previously non-predictive cues* [16].

### 2.4. Statistical Analysis

Patients and controls fulfilling a learning criterion of achieving 6 consecutive correct trials in Stage 1 (patients: *n* = 35; controls: *n* = 41) were included in study analysis and were compared on the number of trials-to-criterion to ensure comparable learning in stage 1 between two groups. Learning scores were analyzed using a 2 × 2 mixed analysis of variance (ANOVA) to examine the LIrr effect, with cue type (predictive vs. nonpredictive) as within-subject variable, group (patients vs. controls) as between-subject variable. Critically, planned *t*-tests were conducted in each group to compare learning scores for predictive and nonpredictive cues, testing for the presence of the LIrr effect in each group. Correlation analyses were then performed to assess relationships of learning scores with various symptom dimensions, antipsychotic dose and cognitive functions. We specifically examined the hypothesized associations between learning about non-predictive (irrelevant) cues and positive symptoms (measured by PANSS positive symptom, PSYRATS total and subscales scores). Bonferroni correction for multiple comparisons was applied to other correlations that were not hypothesis-driven. The level of statistical significance for all analyses (except non-hypothesis-driven correlations) was set at *p* <0.05.

## 3. Results

### 3.1. Characteristics of the Sample

Demographics, cognitive functions and clinical characteristics of the participants are summarized in Table 1. There were no significant differences in age or gender between patients and controls. In line with previous literature showing generalized cognitive impairment in FES samples [36,37], our patients performed significantly worse in all of the cognitive tests and had lower educational level than controls. Among those participants who met a learning criterion and were included in study analysis, no significant group difference in the number of trials-to-criterion in Stage 1 of the LIrr task was observed (patients: 24.6 [18.0], controls: 22.1 [16.1]; *t*_74_ = 0.63, *p* = 0.529).

### 3.2. Learned Irrelevance Task Performance

**Figure 2:**
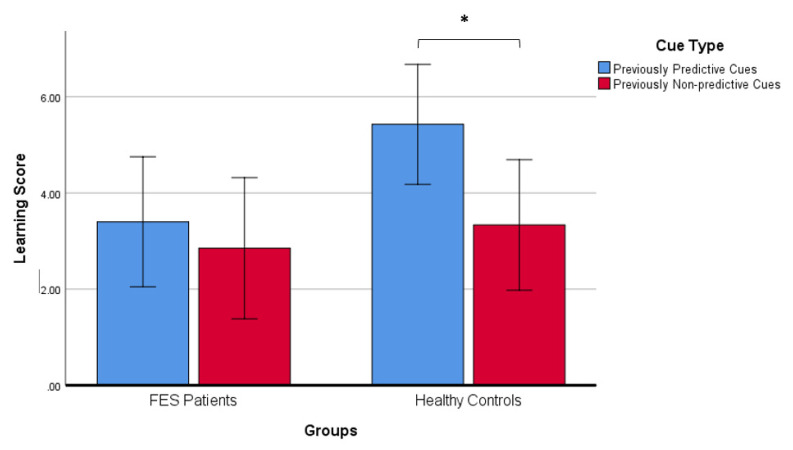
Comparison between learning scores for predictive and non-predictive cues in patients and controls. FES: First-episode schizophrenia. * *p* < 0.05.

Mixed ANOVA revealed a significant main effect of cue-type (*F*_1,74_ = 5.37, *p* = 0.023), but no significant main effect of group (*F*_1,74_ = 2.61, *p* = 0.111) or group by cue-type interaction (*F*_1,74_ = 1.83, *p* = 0.181). Follow-up planned *t*-tests examining the effect of cue-type showed that controls attained significantly higher learning scores for predictive cues than non-predictive cues (*t*_40_ = 2.35, *p* = 0.024), indicating the presence of a LIrr effect in controls (Figure 2). However, no significant difference between learning scores for predictive cues and non-predictive cues was observed among FES patients (*t_34_* = 0.831, *p* = 0.412). That is, patients did not exhibit a LIrr effect, with learning in Stage 2 not discriminating between relevant and irrelevant cues. 

Correlation analyses failed to demonstrate any significant associations between learning scores and various positive symptom measures (Table 2). Learning scores were also not correlated with other symptom domains, antipsychotic dose (in terms of chlorpromazine equivalents) [38] and cognitive functions after correction for multiple comparisons (Table 2 and Table S1). Given that dopamine D2-receptor blockade may modulate the LIrr effect, we also conducted additional partial correlation analyses between learning scores and positive symptom measures, controlling for antipsychotic dose, and revealed lack of significant associations (all *p* values > 0.05). 

## 4. Discussion

The current study sought to investigate dysfunction of selective attention and its relationship with positive symptoms in FES patients using a well-validated LIrr paradigm. Our results affirmed disrupted LIrr in first-episode patients who did not show normal attention bias towards predictive cues over non-predictive cues. This indicates that FES patients failed to distinguish between relevant and irrelevant stimuli. Our findings thus concur with a previous report which employed the same LIrr paradigm and showed lack of an attentional bias towards predictive cues relative to non-predictive cues in schizophrenia patients [16]. This is also consistent with those few prior first-episode studies which revealed absence of a LIrr effect in FES patients using other experimental designs [9,11]. 

Contrary to our hypothesis and the finding of Morris et al. [16] that increased attention to non-predictive cues was correlated with greater positive symptom severity in schizophrenia patients, no such association was observed in our FES sample. Our result is also at odds with another study which found that higher level of positive schizotypy was associated with greater reduction in LIrr effect [39]. It might be possible that our FES patients, who had been briefly treated with antipsychotic medication at the time of testing, exhibited less severe positive symptoms which resulted in reduced variance in symptom ratings as compared to previous studies [16], and hence precluded us from detecting subtle yet potentially significant relationship with attention towards non-predictive cues. Notably, however, our null finding accords with data from two other prior studies which failed to demonstrate significant association between LIrr disruption and positive symptoms in medication-naïve FES patients [9,11], albeit using an LIrr task that was somewhat different from ours, and with small sample size. Likewise, although studies of latent inhibition (LI) have generally demonstrated absence of normal LI during the acute phase of schizophrenia when patients tend to experience more positive symptoms, mixed findings were noted regarding the relationship between impaired LI effect and positive symptom severity [6]. However, owing to the paucity of existing data in this respect, further research is warranted to clarify the association of abnormal allocation of attention to irrelevant stimuli with positive symptoms in the early course of illness. Alternatively, we also explored potential relationships of LIrr performance with other symptom dimensions including negative symptoms, disorganization and depression but showed lack of significant associations. This is nonetheless largely in keeping with findings of earlier studies which failed to consistently demonstrate significant relationships between LIrr measures and ratings of these symptom dimensions in first-episode samples [11,17]. Our results of the absence of significant correlations between LIrr measures and cognitive functions indicate that aberrant LIrr in FES patients could not be explained by their cognitive impairment.

Several methodological limitations warrant consideration in interpreting the study results. First, as the majority of our FES patients were treated with antipsychotic medications before study assessment, we cannot rule out an influence of dopamine D2-receptor blockade on the LIrr effect. Nonetheless, we did not find any significant correlations between antipsychotic dose and LIrr learning scores even when the effect of chlorpromazine equivalents was adjusted. Second, our sample size was modest, albeit the largest compared with previous FES studies on LIrr, and may, therefore, compromise our ability to detect subtle but significant associations. Additionally, a higher control-to-patient ratio, such as 1:2 or 1:3, would further increase the statistical power to identify potential between-group difference in LIrr task performance. Third, the current study was cross-sectional in design. Prospective investigation of LIrr prior to and following antipsychotic initiation in FES patients of adequate sample size is required to elucidate whether loss of normal retarded LIrr, indexing selective attentional deficit with impaired ability to differentiate between relevant and irrelevant stimuli, is a state effect and could be reinstated at clinical status of remission from first psychotic episode by antipsychotic treatment. 

## 5. Conclusions

In conclusion, our results indicate aberrant LIrr in FES patients who fail to exhibit normal attentional bias towards relevant over irrelevant cues. However, significant association between such selective attentional impairment and positive symptom severity could not be demonstrated in the current study. Further research is thus required to clarify the longitudinal trajectory of abnormal allocation of attention as well as its relationship with positive symptoms and treatment response in the early course of illness. 

## Figures and Tables

**Table 1:** Demographics, cognitive functions and clinical characteristics of patients and controls.

Variables of Interest ^1^	Patients (*n* = 35)	Controls (*n* = 41)	χ²/*t*	*p*
Demographics				
Age in years	26.8 (7.9)	24.3 (7.5)	1.44	0.154
Female gender	19 (54.3)	19 (46.3)	0.48	0.490
Years of education	12.8 (2.8)	14.2 (2.7)	−2.35	0.021
Cognitive performances				
Letter number span	14.0 (2.7)	17.0 (3.1)	−4.47	<0.001
Digit symbol	9.5 (3.1)	14.6 (2.9)	−7.39	<0.001
Letter cancellation	4.9 (5.4)	2.0 (2.3)	3.12	0.003
Logical memory	9.0 (3.3)	13.5 (3.6)	−5.70	<0.001
Trail making A	36.2 (14.6)	27.1 (6.5)	3.60	0.001
Trail making B	71.2 (29.0)	48.6 (15.2)	4.34	<0.001
Clinical characteristics				
Age at onset	26.3(8.2)	-	-	-
Duration of untreated psychosis, days (median)	114	-	-	-
PANSS positive symptom score ^2^	20.7 (6.2)	-	-	-
PANSS disorganization score ^2^	8.2 (2.1)	-	-	-
PSYRATS total	54.2 (35.7)	-	-	-
PSYRATS auditory hallucination subscale score	25.5 (17.7)	-	-	-
PSYRATS delusion subscale score	29.5 (21.0)	-	-	-
BNSS total	18.6 (11.5)	-	-	-
CDSS total	4.1 (3.8)	-	-	-
Treatment characteristics		-	-	-
Treatment duration in days (median)	22	-	-	-
Use of antipsychotics ^3^	32 (91.4)			
Chlorpromazine equivalents	322.8 (155.8)	-	-	-

BNSS: Brief Negative Symptom Scale; CDSS: Calgary Depression Scale for Schizophrenia; PANSS: Positive and Negative Syndrome Scale; PSYRATS: Psychotic Symptom Rating Scales. ^1^ Data of all variables are presented in mean and SD except gender and use of antipsychotics (number and percentages), and duration of untreated psychosis and treatment duration (median). ^2^ PANSS positive symptom and disorganization scores were derived based on a previous factor-analytic study in early psychosis sample [28]. ^3^ Among 32 patients who were on antipsychotic medication, 31 were treated with second-generation antipsychotic and 1 was treated with first-generation antipsychotic.

**Table 2:** Correlations of learning scores with symptoms and antipsychotic dose in patients ^1^.

	Predictive Cues		Nonpredictive Cues	
Variables	*r_s_*	*p*	*r_s_*	*p*
PANSS positive symptom score	−0.132	0.451	−0.161	0.356
PSYRATS total score	−0.189	0.278	−0.183	0.294
PSYRATS hallucination subscale score	−0.097	0.587	−0.110	0.534
PSYRATS delusion subscale score	−0.157	0.368	−0.176	0.313
PANSS disorganization score	0.025	0.886	−0.015	0.932
BNSS total score	−0.348	0.041 ^2^	−0.158	0.366
CDSS total score	0.087	0.620	−0.187	0.282
Chlorpromazine equivalents	−0.293	0.103	−0.148	0.418

BNSS: Brief Negative Symptom Scale; CDSS: Calgary Depression Scale for Schizophrenia; PANSS: Positive and Negative Syndrome Scale; PSYRATS: Psychotic Symptom Rating Scales. ^1^ Spearman-rank correlation analyses were conducted. In hypothesis-driven correlations (relationships of nonpredictive-cue learning scores with PANSS positive symptom score, PSYRATS. Total and subscale scores), correction to multiple comparisons was not applied. Bonferroni correction was applied to the remaining correlations. ^2^ Correlation between predictive-cue learning scores and BNSS total score did not survive correction for multiple comparisons (corrected *p* value = 0.00313).

## Data Availability

The data presented in this study are available on reasonable request from the corresponding author.

## Supplementary Materials

The following are available online at https://www.mdpi.com/article/10.3390/brainsci11111370/s1, Table S1: correlations of learning scores with cognitive functions in patients and controls.
