# Supplementary material for: Aberrant Learned Irrelevance in Patients with First-Episode Schizophrenia-Spectrum Disorder
Source: Brain Sci. 2021 Oct 20;11(11):1370. doi: 10.3390/brainsci11111370 (PMC8616017; doi:10.3390/brainsci11111370)
Supplement: Supplementary file 1 [file brainsci-11-01370-s001.zip › brainsci-1375242-supplementary.pdf]

Supplementary Materials

Table S1. Correlations of learning scores with cognitive functions in patients and controls.[1]

	Patients		Controls	
Cognitive test	Predictive cues	Non-predictive cues	Predictive cues	Non-predictive cues
Letter number span	0.027	0.136	0.245	0.256
Digit symbol	0.265	0.109	0.212	0.279
Letter cancellation	-0.136	-0.268	-0.033	0.173
Logical memory	0.266	0.023	0.110	0.151
Trail making A	-0.399[2]	-0.217	-0.153	-0.290
Trail making B	-0.269	-0.267	-0.304	-0.333[2]

[1] Spearman-rank correlation coefficients are presented. Bonferroni correction for multiple comparisons was applied.
[2] Correlations between predictive-cue learning scores and Trail making A ($p = 0.018$) in patients, and between nonpredictive-cue learning scores and Trail making B ($p = 0.033$) in controls did not survive correction for multiple comparisons (corrected p value = 0.002).
